# One year of COVID-19: infection rates and symptoms in patients with inherited metabolic diseases followed by MetabERN

**DOI:** 10.1186/s13023-022-02247-3

**Published:** 2022-03-04

**Authors:** Laura Paneghetti, Cinzia Maria Bellettato, Annalisa Sechi, Karolina M. Stepien, Maurizio Scarpa

**Affiliations:** 1 MetabERN, Regional Coordinating Center for Rare Diseases, Udine University Hospital, Udine, Italy; 2 Adult Inherited Metabolic Diseases, Salford Royal NHS Foundation Trust, Salford, UK

**Keywords:** Inherited metabolic disorders, IMDs, COVID-19, SARS-CoV-2

## Abstract

**Background:**

Since the beginning of the COVID-19 pandemic, MetabERN has been monitoring the SARS-CoV-2 infection rates within its metabolic community. To gather data on the total number of cases and the severity of symptoms among IMD patients one year into the pandemic, an online survey was distributed among all MetabERN healthcare providers (HCP). Epidemiological analysis was performed by integrating the survey’s data with the MetabERN database.

**Results:**

Survey’s respondents reported a total of 452 cases of COVID-19 among their IMD patients (213 paediatric and 239 adults). Considering the total number of patients followed by the respondents (n = 26,347), the registered prevalence of COVID-19 in the IMD population was of 1716 × 100,000. Italy emerged as the most affected country (25.4% of cases), followed by the United Kingdom (14.2% of cases). Most of the paediatric cases of COVID-19 displayed no or mild symptoms during the disease: 34% of HCP reported having asymptomatic patients in 75–100% of cases, while 37.5% reported mild symptoms in about a quarter of their patients. Similarly to paediatric cases, most adult IMD patients with COVID-19 were asymptomatic or had mild symptoms: about one third of respondents reported 75–100% asymptomatic patients and about 65% of HCP had between 0 and 50% of patients with mild symptoms. The majority of the respondents reported no deaths due to COVID-19 in adult and paediatric patients with IMDs.

**Conclusions:**

Most of MetabERN’s IMD patients who got COVID-19 during the first year of the pandemic had mild symptoms and a positive outcome of the disease. However, fatal events were recorded in paediatric patients; this, together with the lack of information on the long-term effects of COVID-19 in IMDs, call for caution in the metabolic population.

**Supplementary Information:**

The online version contains supplementary material available at 10.1186/s13023-022-02247-3.

## Background

Emerged in December 2019 in China, the Coronavirus disease 2019 (COVID-19) caused by the severe acute respiratory syndrome coronavirus 2 (SARS-CoV-2) is still a global pandemic. As of 4^th^ October 2021, there have been 234,809,103 confirmed cases worldwide; of these, almost 71 million have been in Europe [1]. Various vaccines to prevent COVID-19 have been approved by the European Medicines Agency [2], and their distribution among the European population is progressing at good rates, with 79% of the adult population in EU/EEA having received the first dose and 73.5% being fully vaccinated as of 4th October 2021 [3]. However, in 2021 new variants of the SARS-CoV-2 have spread and raised concerns; among these, the variant B.1.1.7 (alpha), first identified in the United Kingdom, appears to be more transmissible than previous strains and may cause more severe infection, while the variant B.1.617.2 (delta), detected at first in India, shows an even higher transmissibility [4]. These aspects are complicating the situation globally.

The European Reference Network for Hereditary Metabolic Disorders (MetabERN), representing 78 healthcare providers (HCP) from 23 EU Member States and 41 patients’ organizations (PO), follows almost 33,000 patients with rare inherited metabolic disorders (IMD). Since the beginning of the pandemic, the network has been monitoring the SARS-CoV-2 infection rates within its metabolic community. Data gathered between March and April 2020, at the beginning of the emergency, showed that the incidence of COVID-19 in the European IMD community was of 72.9 × 100,000 for paediatric and adult cases, which was lower than that of the general European population (EU/EEA and UK) reported from 1st January to 7th April 2020 (117 × 100,000) [5]. Thanks to local restrictions, emergency protocols put into place by HCP, and specific information shared with the patients, these data suggested that the network of HCP and PO was able to protect metabolic patients from SARS-CoV-2 infection, at least in part, during the first wave [5].

To our knowledge, at present there is no data available regarding the frequency of COVID-19 among IMD patients and the severity and consequences of the disease in metabolic patients. To gather this type of information, MetabERN distributed a dedicated survey among its members one year into the pandemic; here we present the results of the survey.

## Results

Sixty metabolic experts distributed in 50 centres across 21 European countries and following a total of 26,347 IMD patients replied to our survey. Of these HCP, 11.7% care for adult patients, 30% follow paediatric cases, while 58.3% follow both (Table 1). Each center usually cares for IMD patients with different types of conditions; the most represented in our survey were (1) lysosomal storage disorders (LSD), (2) carbohydrate, fatty acid oxidation and ketone bodies disorders (C-FAO), (3) amino and organic acids-related disorders (AOA), and (4) disorders of pyruvate metabolism, mitochondrial oxidative phosphorylation, thiamine transport and metabolism, and Krebs cycle defects (PM-MD), which are treated by 85%, 77%, 75% and 72% of respondents, respectively (Table 1).

**Table 1:** Groups of patients and IMD types followed by MetabERN’s HCP and affected by COVID-19

Category	n (%)
Group of IMD patients followed at the centre	
Adult	7/60 (11.7)
Paediatric	18/60 (30)
Both	35/60 (58.3)
IMD types that affect the patients under the respondent’s care^a^	
LSD	51/60 (85)
C-FAO	46/60 (76.7)
AOA	45/60 (75)
PM-MD	43/60 (71.7)
CDG	38/60 (63.3)
NOMS	25/60 (41.7)
PD	25/60 (41.7)
Since the beginning of the pandemic, have you had any paediatric IMD patients confirmed positive for SARS-CoV-2 by either antigen, molecular or antibody testing?	
Yes	34/60 (56.7)
No	22/60 (36.7)
Do not know	4/60 (6.7)
No answer	0/60 (0)
Among your paediatric patients who tested positive for COVID-19, which IMD types were mainly affected?^a^	
AOA	19/34 (55.9)
LSD	13/34 (38.2)
C-FAO	11/34 (32.4)
CDG	5/34 (14.7)
PM-MD	2/34 (5.9)
PD	0/34 (0)
NOMS	0/34 (0)
No answer	2/34 (5.9)
Since the beginning of the pandemic, have you had any adult IMD patients confirmed positive for SARS-CoV-2 by either antigen, molecular or antibody testing?	
Yes	26/60 (43.3)
No	19/60 (31.7)
Do not know	13/60 (21.7)
No answer	2/60 (3.3)
Among your adult patients who tested positive for COVID-19, which IMD types were mainly affected?^a^	
LSD	18/26 (69.2)
AOA	12/26 (46.2)
C-FAO	9/26 (34.6)
PM-MD	4/26 (15.4)
PD	1/26 (3.9)
CDG	1/26 (3.9)
NOMS	1/26 (3.9)
No answer	0/26 (0)

*AOA* amino and organic acids-related disorders, *PM-MD* disorder of pyruvate metabolism, Krebs cycle defects, mitochondrial oxidative phosphorylation disorders, disorders of thiamine transport and metabolism, *C-FAO* carbohydrate, fatty acid oxidation and ketone bodies disorders, *LSD* lysosomal storage disorders, *PD* peroxisomal disorders, *CDG* congenital disorders of glycosylation and disorders of intracellular trafficking, *NOMS* disorders of neuromodulators and other small molecules

^a^More than one answer possible

**Fig. 1:**
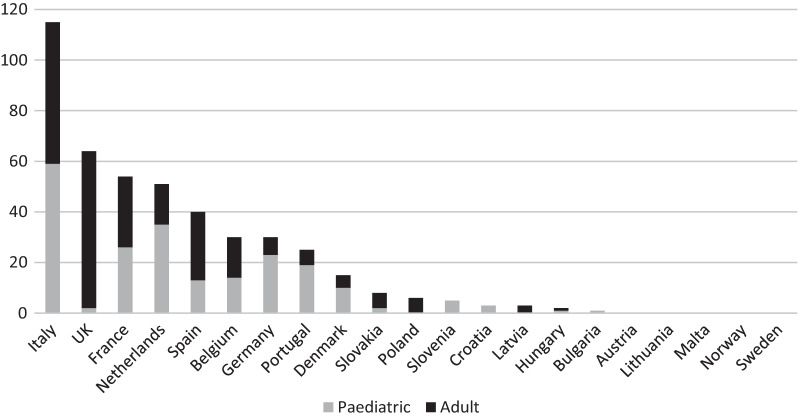
COVID-19 cases among MetabERN’s IMD patients per country

Survey participants reported a total of 452 cases of COVID-19 among their IMD patients since the beginning of the pandemic: 213 paediatric and 239 adults. Considering that total number of patients followed by the respondents (n = 26,347), the registered prevalence of COVID-19 in the IMD population of reference is of 1716 × 100,000. With a total of 115 cases (25.4%), Italy emerged as the most affected country, followed by the United Kingdom (64 cases; 14.2%) (Table 2 and Fig. 1).

**Table 2:** Number of COVID-19 cases reported by MetabERN’s HCP

Country	Paediatric	Adult	Total	%	Responding centres^a^	Tot number of centres^a^/country
Italy	59	56	115	25.4	8	11
UK	2	62	64	14.2	2	6
France	26	28	54	11.9	5	9
Netherlands	35	16	51	11.3	3	5
Spain	13	27	40	8.8	3	5
Belgium	14	16	30	6.6	5	6
Germany	23	7	30	6.6	6	9
Portugal	19	6	25	5.5	4	5
Denmark	10	5	15	3.3	1	1
Slovakia	2	6	8	1.8	1	1
Poland	0	6	6	1.3	1	1
Slovenia	5	0	5	1.1	1	1
Croatia	3	0	3	0.7	1	1
Latvia	0	3	3	0.7	1	1
Hungary	1	1	2	0.4	1	1
Bulgaria	1	0	1	0.2	1	1
Austria	0	0	0	0.0	1	2
Lithuania	0	0	0	0.0	1	1
Malta	0	0	0	0.0	1	1
Norway	0	0	0	0.0	3	2
Sweden	0	0	0	0.0	1	2
Total	213	239	452	100%	50	72

^a^Centres belonging to the MetabERN network

**Fig. 2:**
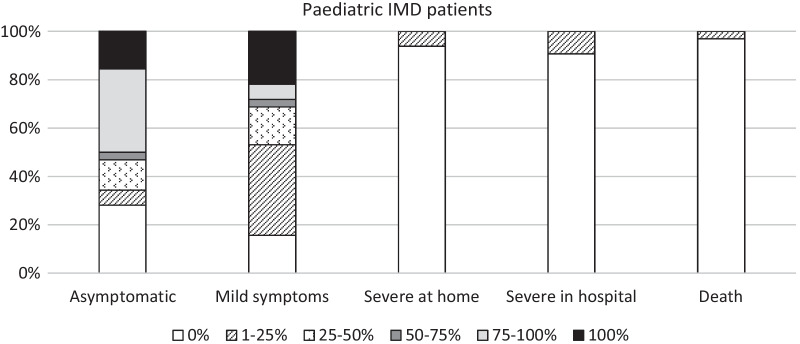
severity of COVID-19 symptoms reported by MetabERN’s HCP in paediatric IMD patients

The majority of respondents (56.7%) have had cases of SARS-CoV-2 infection among their paediatric patients (Table 1); a high number of these were in patients affected by AOA (59.4%), followed by LSD (40.6%) and C-FAO (34.4%) (Table 1). Most of the paediatric cases of COVID-19 displayed no or mild symptoms during the disease: 34% of respondents said that their patients were asymptomatic in 75–100% of cases, while 37.5% HCP reported mild symptoms in about a quarter of their patients (Fig. 2). The number of severe cases of COVID-19 was negligible: less than 10% of respondents said that 1–25% of their patients had severe symptoms or required hospitalisation (Fig. 2). Importantly, 97% of HCP had no fatalities among their IMD patients affected with COVID-19; however, 3% of HCP reported deaths in 1–25% of infected patients (Fig. 2). In most of these patients, death was directly related to the viral infection, with some patients having the COVID-19 superimposed on their slowly progressing neurodegenerative condition (personal observations from clinical experience of the authors).

**Fig. 3:**
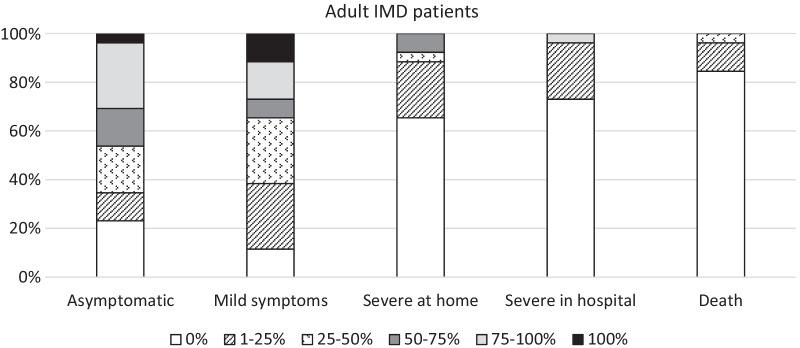
severity of COVID-19 symptoms reported by MetabERN’s HCP in adult IMD patients

SARS-CoV-2 infection among adult IMD patients was reported by 44.8% of respondents (Table 1). The significant proportion of these cases were patients with LSD (69.2%), AOA (46.2%) and C-FAO (34.6%) (Table 1). Similarly to paediatric cases, most adult IMD patients who got COVID-19 were asymptomatic or had mild symptoms: about one third of respondents reported 75–100% asymptomatic patients and about 65% of HCP had between 0 and 50% of patients with mild symptoms (Fig. 3). However, 23% of respondents also reported about 1–25% of cases developing severe symptoms and managing either at home or requiring hospitalisation, while about 8% of HCP said that 50–75% of their COVID-19 patients were severe but were not hospitalised (Fig. 3). Compared to paediatric cases, COVID-19-related deaths in adult IMD patients were slightly higher: 11.5% of respondents said that 1–25% of their patients have died, and for almost 4% of HCP this percentage was 25–50% (Fig. 3). Nonetheless, the majority of respondents (84.6%) report no deaths due to COVID-19 in adult patients with IMDs (Fig. 3).

## Discussion

At the time the survey was concluded (end of March 2021) the ECDC reported 27,570,251 cumulative cases of COVID-19 in the EU/EEA region since January 2020 [6], equal to a prevalence of 6094 × 100,000 in the general population of Europe. Compared to this rate and based on the data presented in this paper, the prevalence we recorded in MetabERN’s IMD population was much lower (1716 × 100,000). This observation may be due to the fact that IMD patients’ and their caregivers’ perception of being at risk of COVID-19-related complications probably prompt them to be keener than the general population in respecting preventative measures like social distancing, increased hand washing, mask wearing, and decreased social interactions and activities [7, 8]. As a result, patients with IMD continued to shield throughout most part of 2020 and 2021. Moreover, as mentioned in a previous publication [5], the low prevalence of COVID-19 among IMD patients may be the result of a solid network of HCPs and patient organizations that worked together to effectively protect metabolic patients from SARS-CoV-2 infection by implementing telemedicine tools and home therapy, where available, or by shifting the workload to the primary care setting [9]. Lastly, this could also be the result of MetabERN’s recommendations on COVID-19, which have been largely and effectively disseminated inside the network [10]

The country that reported most cases of COVID-19 among IMD patients was Italy. This may be due to the fact that Italy is the EU country with one of the highest rates of COVID-19 infections; another reason for this result is that Italy was the country that participated the most to the survey (eight centres in total).

In our survey, most paediatric and adult IMD patients affected with COVID-19 were known to have an underlying metabolic disorder such as AOA, LSD, or C-FAO. These disorders are generally considered at high risk of metabolic decompensation, cardio-respiratory complications, and frequent exacerbation induced by infections, so they may be more vulnerable also to COVID-19-induced complications [8]. Most of the survey respondents are aware that viral infection may instigate acute metabolic decompensation, which results in Intensive Care Unit admission, requires prolonged in-hospital stay, and often haemofiltration and the use of intravenous medications. The prognosis of such complex cases with several risk factors can be poor. However, we cannot say for certain that patients with AOA, LSD, or C-FAO have a higher risk of severe acute metabolic illness or other viral-related complications when infected with SARS-CoV-2. Indeed, according to our data, most IMD patients with COVID-19 (both paediatric and adults) had no or mild symptoms.

In line with the general population and compared to the paediatric patients [11], adults with an IMD who contracted SARS-CoV-2 infection experienced more severe symptoms, required hospitalisation or died from COVID-19-related complications. Based on the available evidence, we cannot say that compared to the general population IMD patients were more “protected” from COVID-19, because they contracted the infection and there were some cases that resulted in death, even among paediatric cases. At the same time, at this stage we do not have enough data to say whether IMD patients are at a higher, lower or equal risk of COVID-19-related complications compared to the general population; more studies are needed to clarify this aspect.

Since the pathway of SARS-CoV-2 infection involves lysosomes trafficking for egress [12], some authors have hypothesized that LSDs such as Niemann Pick type C and Gaucher disease are less at risk of SARS-CoV-2 infection or severe COVID-19 complications [13, 14]. Our data demonstrate that LSDs are not immune towards COVID-19; on the contrary they were the largest group of the metabolic disorders in our adult casualty list. While this result is probably due to the fact that most of the survey respondents are specialised in LSDs and follow this type of patients, due to the limitations of the survey we cannot add any further comment regarding the specific LSD conditions.

This study has several limitations. Firstly, the data presented is limited to 50 out of 78 centres belonging to the MetabERN network, so we cannot draw conclusions regarding the entire MetabERN network or the overall IMD population in Europe and around the world. Secondly, the reported number of affected patients is certainly underestimated (low case detection) as not all centers in the MetabERN network contributed with their data. In addition, not all IMD patients followed by the network were tested for COVID-19: only symptomatic cases, those with a family member affected with COVID-19 or those that require investigations such as a pulmonary function test are usually tested; also, it is possible that some patients were affected with a mild infection that did not require hospitalization and felt like it was not necessary for them to report it to their metabolic physician. Furthermore, IMD patients who developed complications of COVID-19 might have had other comorbidities such as diabetes mellitus, increased body mass index, or were of certain ethnic origin (BAME). These factors might have impacted the course of their illness and their recovery from it. The detailed information regarding the above was not collected in the survey. Lastly, in MetabERN’s centers there is a high representation of LSD patients, which does not accurately reflect the reality of IMD patients in Europe. Given the vast differences in local epidemiology and policy interventions and the uneven geographical distribution of MetabERN HCP across each country and across the EU, more time and data are needed to determine the actual consequences of the COVID-19 pandemic on the IMD population.

Many changes in hospital visits and disease management were implemented at the very beginning of the pandemic to protect patients, especially because experts thought that IMD patients were at a higher risk to develop severe forms of COVID-19 [5]. In April 2020, MetabERN published its recommendations for all IMD patients and caregivers about treatment adherence during the emergency [8]. In March 2021, the network published additional recommendations for vaccination based on the available data, which suggested that infections could start a metabolic decompensation in IMDs such as AOA, PM-D, C-FAO or LSD, and put patients at risk of metabolic decompensation, respiratory or cardiac complications, and frequent exacerbation [8]. The vaccination among the paediatric population is still a matter of debate worldwide. The fatal events in some paediatric patients reported in our study prompt MetabERN to recommend the inclusion of paediatric IMD patients as a priority group for COVID-19 vaccination. There is still a lot unknown about the long-term effects of COVID-19 in the general population and especially in metabolic patients. These survey results warrant further research into the impact of COVID-19 on the IMD population.

## Conclusions

During the first year of COVID-19 pandemic, the majority of MetabERN’s IMD patients who got the disease experienced mild symptoms and had a positive outcome. However, the fatal events recorded among paediatric patients together with the limited knowledge regarding the long-term effects of COVID-19 call for caution and special attention in the metabolic population. More time is needed to perform further studies on the topic; in the meantime, IMD paediatric patients should be included as a priority group for COVID-19 vaccination.

## Methods

The Survey Monkey platform was used to design and distribute the survey and collect data. The survey included 12 questions, was distributed to all MetabERN centers and was active between 20 and 29th March 2021 (Additional file 1). The data were extracted and analysed with Microsoft Excel. Epidemiological analysis was performed by integrating the survey’s data with the MetabERN database, which includes the number of adult and paediatric patients followed by each HCP member of the network, where a patient is considered an adult when he/she reaches the age of 18 years. For the statistical analysis, the total number of patients followed by the centres that responded to the survey (n = 26,347) was used as the population of reference.

## Supplementary Information

**Additional file 1.** Original survey questionnaire.

## Data Availability

The datasets used and/or analysed during the current study are available from the corresponding author on reasonable request.

## Abbreviations

IMDs: Inherited metabolic disorders
HCP: Healthcare provider
MetabERN: European Reference Network for Rare Hereditary Metabolic Disorders
AOA: Amino and organic acids-related disorders
PM-MD: Disorder of pyruvate metabolism, Krebs cycle defects, mitochondrial oxidative phosphorylation disorders, disorders of thiamine transport and metabolism
C-FAO: Carbohydrate, fatty acid oxidation and ketone bodies disorders
LSD: Lysosomal storage disorders
PD: Peroxisomal disorders
CDG: Congenital disorders of glycosylation and disorders of intracellular trafficking
NOMS: Disorders of neuromodulators and other small molecules
