# Supplementary material for: One year of COVID-19: infection rates and symptoms in patients with inherited metabolic diseases followed by MetabERN
Source: Orphanet J Rare Dis. 2022 Mar 4;17:109. doi: 10.1186/s13023-022-02247-3 (PMC8894822; doi:10.1186/s13023-022-02247-3)
Supplement: Supplementary file 1 — Additional file 1. Original survey questionnaire. [file 13023_2022_2247_MOESM1_ESM.pdf]

Introduction

While we are still dealing with the second wave of the coronavirus pandemic, we have designed this new survey on COVID-19 to have a complete overview of the current status of our IMD community and develop further publications.

In particular, we think it is important to update our data on the number of infections and the severity of symptoms in IMD patients to better understand the impact of COVID-19 on our patients.

The estimated time to complete the whole survey is 5 minutes.

* 1. Please fill in your name, country, and email address.

Name ________

Country ________

Email Address ________

* 2. Please select the name of your hospital /institute from the drop-down menu:

* 3. Please select your group of patients:

- ◯ Adult
- ◯ Paediatric
- ◯ Both

* 4. Please select the IMD types that affect the patients who are under your care:

- ☐ Amino and organic acids-related disorders (AOA)
- ☐ Disorder of pyruvate metabolism, Krebs cycle defects, mitochondrial oxidative phosphorylation disorders, disorders of thiamine transport and metabolism (PM-MD)
- ☐ Carbohydrate, fatty acid oxidation and ketone bodies disorders (C-FAO)
- ☐ Lysosomal storage disorders (LSD)
- ☐ Peroxisomal disorders (PD)
- ☐ Congenital disorders of glycosylation and disorders of intracellular trafficking (CDG)
- ☐ Disorders of Neuromodulators and Other Small Molecules (NOMS)

* 5. Since the beginning of the pandemic, have you had any **paediatric** IMD patients confirmed positive for SARS-CoV-2 in your centre by either antigen or antibody testing?

- ◯ Yes
- ◯ No
- ◯ I don't know

COVID-19: update on infection rate and symptoms in IMD patients

Paediatric cases

* 6. Since the beginning of the pandemic, how many paediatric cases of COVID-19 did you have? (To select a number slide the bar below)

0 100

* 7. Among your paediatric patients who tested positive for COVID-19, which IMD types were mainly affected?

- ☐ Amino and organic acids-related disorders (AOA)
- ☐ Disorder of pyruvate metabolism, Krebs cycle defects, mitochondrial oxidative phosphorylation disorders, disorders of thiamine transport and metabolism (PM-MD)
- ☐ Carbohydrate, fatty acid oxidation and ketone bodies disorders (C-FAO)
- ☐ Lysosomal storage disorders (LSD)
- ☐ Peroxisomal disorders (PD)
- ☐ Congenital disorders of glycosylation and disorders of intracellular trafficking (CDG)
- ☐ Disorders of Neuromodulators and Other Small Molecules (NOMS)

* 8. During the course of COVID-19, your paediatric IMD patients (please quantify % of cases):

	0%	1-25%	25-50%	50-75%	75-100%	100%
Were asymptomatic	○	○	○	○	○	○
Displayed mild symptoms	○	○	○	○	○	○
Displayed severe symptoms but managed at home (e.g. required oxygen)	○	○	○	○	○	○
Displayed severe symptoms and were hospitalised	○	○	○	○	○	○
Required intensive care	○	○	○	○	○	○
Died	○	○	○	○	○	○

COVID-19: update on infection rate and symptoms in IMD patients

Adult cases

* 9. Since the beginning of the pandemic, have you had any **adult** IMD patients confirmed positive for SARS-CoV-2 in your centre by either antigen or antibody testing?

- ○ Yes
- ○ No
- ○ I don't know

COVID-19: update on infection rate and symptoms in IMD patients

Adult cases

* 10. Since the beginning of the pandemic, how many adult cases of COVID-19 did you have? (To select a number slide the bar below)

0 100

* 11. Among your adult patients who tested positive for COVID-19, which IMD types were mainly affected?

- [ ] Amino and organic acids-related disorders (AOA)
- [ ] Disorder of pyruvate metabolism, Krebs cycle defects, mitochondrial oxidative phosphorylation disorders, disorders of thiamine transport and metabolism (PM-MD)
- [ ] Carbohydrate, fatty acid oxidation and ketone bodies disorders (C-FAO)
- [ ] Lysosomal storage disorders (LSD)
- [ ] Peroxisomal disorders (PD)
- [ ] Congenital disorders of glycosylation and disorders of intracellular trafficking (CDG)
- [ ] Disorders of Neuromodulators and Other Small Molecules (NOMS)

* 12. During the course of COVID-19, your adult IMD patients (please quantify % of cases):

	0%	1-25%	25-50%	50-75%	75-100%	100%
Were asymptomatic	○	○	○	○	○	○
Displayed mild symptoms	○	○	○	○	○	○
Displayed severe symptoms but managed at home (e.g. required oxygen)	○	○	○	○	○	○
Displayed severe symptoms and were hospitalised	○	○	○	○	○	○
Died	○	○	○	○	○	○
